# Development and Validation of a Tool for Assessing Glucose Impairment in Adolescents

**DOI:** 10.5888/pcd911_0213

**Published:** 2012-05-24

**Authors:** Katrina D. DuBose, Doyle M. Cummings, Satomi Imai, Suzanne Lazorick, David N. Collier

**Affiliations:** Author Affiliations: Doyle M. Cummings, Satomi Imai, Suzanne Lazorick, David N. Collier, East Carolina University, Greenville, North Carolina.

## Abstract

**Introduction:**

Childhood obesity is associated with an increased risk for type 2 diabetes. Early identification of adolescents at risk for impaired fasting blood glucose may lead to earlier and more comprehensive evaluation and intervention. Because widespread blood glucose testing of adolescents is not recommended, community-based tools are needed to identify those who could benefit from further testing. One such tool, developed for adults, was the Tool for Assessing Glucose ImpairmenT (TAG-IT). Our objective was to validate whether a similar tool could be useful for community-based screening of glucose impairment risk among adolescents.

**Methods:**

Our study sample consisted of 3,050 adolescents aged 12 to18 years who had participated in the 1999-2008 National Health and Nutrition Examination Survey (NHANES). Half of participants were female and 40% were nonwhite. NHANES measured fasting blood glucose and height, weight, and resting heart rate. We used Pearson correlations and regression analysis to determine key variables for predicting glucose impairment. From these measurements, we created a composite TAG-IT score for adolescents called TAG-IT-A. We then applied the TAG-IT-A model to 1988-1994 NHANES data, using linear regression analysis and receiver operating characteristic analysis to determine how well the TAG-IT-A score predicted a fasting blood glucose at or above 100 mg/dL.

**Results:**

We determined that age, sex, body mass index, and resting heart rate were predictors of impaired fasting blood glucose and that TAG-IT-A was a better predictor of impaired fasting blood glucose than body mass index alone (area under the curve, 0.61, *P* < .001 vs 0.55, *P* = .10, respectively). A TAG-IT-A score of 3 or higher correctly identified 50% of adolescents with impaired fasting blood glucose, while a score of 5 or higher correctly identified 76% .

**Conclusion:**

The TAG-IT-A score is a simple screening tool that clinicians and public health professionals could use to easily identify adolescents who may have impaired fasting blood glucose and need a more comprehensive evaluation.

## Introduction

Obesity rates have tripled in the past 20 years among children and adolescents, leading to an obesity epidemic in this population (1,2). Concurrent with this increase in obesity has been an increase in the incidence of type 2 diabetes in this same population (3). An estimated 1 in 3 children born in the United States in 2000 will develop type 2 diabetes at some point in their lifetime (4).

Because few children have impaired fasting blood glucose, widespread use of glucose screening in this population is not recommended. In addition, many adolescents seek health care infrequently and only for acute problems and thus may go years without contact with a health care provider. New tools are needed for community-based screening to identify those at increased risk of glucose impairment and in need of further evaluation or interventions.

In adults, the Tool for Assessing Glucose ImpairmenT (TAG-IT) was developed for use in the community to assess risk of glucose impairment (5). Compared with using body mass index (BMI) alone, TAG-IT was much better at predicting glucose impairment. Furthermore, a TAG-IT score of 5 or higher correctly identified adults with glucose impairment 87% of the time (5). In addition to TAG-IT, He et al (6) developed the Abnormal Glucose Risk Asessment-6 (AGRA-6), which includes 6 models for assessing risk for abnormal glucose levels. These models examine different measures of abnormal glucose, such as impaired fasting blood glucose, impaired glucose tolerance, undiagnosed diabetes, and all other types of glucose impairment. The area under the curve for all 6 models was 0.72 to 0.80. The authors also reported that the AGRA-6 models accurately predicted abnormal blood glucose in about 70% of adults (6).

Although both TAG-IT and AGRA-6 are valid community screening tools to assess glucose impairment risk in adults (5,6), to our knowledge, a similar glucose impairment screening tool for adolescents (aged 12-18 y) does not exist. Adolescents are growing and transitioning through puberty, which may affect body composition, physical activity levels, resting heart rate, and insulin resistance. Therefore, methods for predicting insulin resistance or glucose impairment in adults may not be effective in adolescents.

Our objective was to develop and validate a community-based screening tool specifically for adolescents. The tool is based on TAG-IT, the adult screening tool, and is called Tool for Assessing Glucose ImpairmenT among Adolescents (TAG-IT-A). We hypothesized that the TAG-IT-A tool would effectively predict risk of impaired fasting blood glucose levels in adolescents.

## Methods

The Centers for Disease Control and Prevention (CDC) conducts the National Health and Nutrition Examination Survey (NHANES) annually. NHANES uses complex probability sampling with a cluster sample design to assess the health and nutritional status of adults and children in the United States. For our study, we used multiple NHANES data sets. We used the 1999-2008 NHANES data to develop TAG-IT-A because NHANES oversampled adolescents and racial/ethnic minorities during these years to provide more precise population estimates. Furthermore, given the rise in the prevalence of type 2 diabetes, there were more adolescents with elevated glucose levels in later years (1999-2008) compared with earlier NHANES data sets (1988-1994). After using the 1999-2008 NHANES data to develop TAG-IT-A, we used NHANES III (1988-1994) data to validate the TAG-IT-A score in a manner similar to that described by Koopman et al. (5). The NHANES protocols were approved by CDC’s institutional review board. We obtained written informed consent from legal guardians and assent from minors.

### TAG-IT-A development sample

To develop the TAG-IT-A score, we used data from adolescents aged 12 to 18 who participated in the fasting subsample of the NHANES 1999-2008 survey. We excluded participants who did not have a fasting blood sample, were currently taking diabetes medication, or had incomplete data on key variables used in the data analysis.

### TAG-IT-A variables

The purpose of the screening tool is to use demographic and noninvasive clinical variables to identify adolescents with a fasting blood glucose at or greater than 100 mg/dL, defined by the American Diabetes Association as impaired (7). Key variables used in the development of the screening tool for adolescents were modeled after those used to develop the adult screening tool, TAG-IT (5). The variables considered for inclusion in the adolescent screening tool were age, race, sex, body mass index (BMI), resting heart rate, hypertension diagnosis (measured resting blood pressure or physician diagnosis), and fasting blood glucose. The only variable investigated in the adult tool that was not explored with the adolescent screening tool was a family history of diabetes, because NHANES did not ask adolescents about this.

Age, race, and sex were all self-reported, and race was categorized as non-Hispanic white, non-Hispanic black, Hispanic, or other (Asian and American Indian). Age was then divided into 2 groups, 12 to 14 and 15 to 18. BMI was calculated from height and weight, which was measured by trained technicians who used standard NHANES methods (8). We used BMI values from the CDC growth charts for US children to categorize each adolescent as normal weight (≤84th percentile), overweight (85th-94th percentile), or obese (≥95th percentile) (9). Resting heart rate and blood pressure were measured by using standard NHANES procedures (10). Resting heart rate was grouped into 10-beat intervals (eg, <60, 60-69, 70-79.). We classified hypertension as a resting blood pressure at or greater than the 95th percentile adjusted for age, sex, and height or participant self-report of a physician diagnosis of hypertension (11).

### TAG-IT-A validation sample

To validate TAG-IT-A we used data on adolescents who participated in the fasting blood glucose subsample of NHANES III (1988-1994). We excluded adolescents who did not have a fasting blood sample, were currently taking diabetes medication, or had incomplete data on key variables used in the data analysis. The same variables used to develop the TAG-IT-A tool were used in the validation sample. Age, race, sex, and BMI were used as the predictive variables of the population from the NHANES III database, and fasting blood glucose was used as the outcome variable to validate the TAG-IT-A score.

### Statistical analysis

In all analyses, we used NHANES weights to accommodate the complex sampling design of NHANES. The weights account for geographic probability sampling methods and oversampling of specific groups, such as minorities and adolescents, and are designed to ensure that the weighted findings are representative of all US adolescents.

We used scatter plots to examine the relationship between fasting blood glucose levels and age, race, sex, BMI, resting heart rate, and hypertension. Pearson product moment and point biserial correlations were used to examine the relationships among these key variables. Following the approach taken by Koopman et al (5) in adults, we used multiple regression analysis to identify the variables associated with a fasting blood glucose of 100 mg/dL or higher. Potential independent variables that we entered into the regression model were age, race, sex, BMI, resting heart rate, and hypertension diagnosis; fasting blood glucose was the dependent variable entered. If the *P* value was less than or equal to .05, the variable was associated with an impaired fasting blood glucose level. Once we identified significant variables, we used logistic regression analysis to examine the relationship of these variables to fasting blood glucose levels. We also used the logistic regression model to generate weighted scores for each key factor that the TAG-IT-A score comprised. The method for developing the weighted scores was modeled after the widely used Charlson Comorbidity Index (12), in which odds ratios (ORs) from 1.0 to 1.19 were assigned 0 points, ORs from 1.2 to 1.49 were assigned 1 point, ORs from 1.5 to 2.49 were assigned 2 points, and so on. After the TAG-IT-A scoring system was created, receiver operating characteristic (ROC) curves were constructed to examine the area under the curve for TAG-IT-A’s ability to predict fasting blood glucose impairment in the NHANES III sample. For comparison, an ROC curve was also generated to examine the ability of BMI alone to predict fasting blood glucose impairment in the NHANES III sample. We used SAS-callable SUDAAN version 9 (RTI International, Research Triangle Park, North Carolina) to conduct all data analysis except for ROC generation, for which we used SAS version 9 (SAS Institute, Inc, Cary, North Carolina). We set statistical significance at *P* < .05.

## Results

A total of 3,050 adolescents from NHANES 1999-2008 with complete data were included in the analysis to develop TAG-IT-A. Most participants were white (63%); 51% were male, 32% were either overweight or obese, and 16% had a fasting blood glucose value of 100 mg/dL or higher (Table 1). A total of 2,564 adolescents from the NHANES III sample were analyzed for validation of TAG-IT-A. The sample was primarily white (66%); 51% were male, 27% were either overweight or obese, and 11% had a fasting blood glucose value of 100 mg/dL or higher.

**Table 1:** Characteristics Included in Development of TAG-IT-A, NHANES, 1999-2008

Characteristic	Proportion of Weighted Sample, %^a^	Number in Weighted Sample (Millions)^a^
**Age, y**		
12-14	41.4	1.2
15-18	58.6	1.7
**Race/ethnicity**		
Non-Hispanic white	62.7	17.9
Non-Hispanic black	14.4	4.1
Hispanic	16.6	3.1
Other	6.2	1.8
**Sex**		
Female	49.0	14.0
Male	50.9	14.6
**Fasting blood glucose, mg/dL**		
100-125	15.6	4.5
≥126	0.2	0.5
**BMI percentile for age and sex^b^**		
≤84th (normal)	67.7	19.2
85th-94th (overweight)	14.8	4.2
≥95th (obese)	17.5	4.9
**Resting heart rate, beats per minute**		
<60	7.5	2.2
60-69	23.8	6.8
70-79	30.1	8.6
80-89	23.7	6.8
≥90	15.0	4.3
**Hypertension^c^**		
No	97.2	2.3
Yes	2.8	0.8

Abbreviations: TAG-IT-A, Tool for Assessing Glucose ImpairmenT among Adolescents; NHANES, National Health and Nutrition Examination Survey; BMI, body mass index.

^a^ Based on weighted population estimates derived by using SUDAAN version 9 (Research Triangle Institute, Research Triangle Park, North Carolina) and NHANES population weights.

^b^ Based on Centers for Disease Control and Prevention growth charts (9).

^c^ Hypertension was defined as ≥95th percentile adjusted for age, sex, and height or physician-diagnosed hypertension.

Correlations among the variables examined for the TAG-IT-A score were low, but we found a positive and significant relationship between fasting blood glucose, age, BMI, and resting heart rate (Table 2). Age was positively related to BMI but negatively related to resting heart rate. Sex was positively related to BMI and resting heart rate. Finally, BMI was positively related to hypertension status.

**Table 2:** Correlations Among Key Variables for TAG-IT-A

Variable	*r* (*P* Value)					
	Fasting Blood Glucose	Age	Sex	Body Mass Index	Resting Heart Rate	Hypertension
Fasting blood glucose	1					
Age	.07 (<.001)	1				
Sex	.23 (<.001)	−.02 (.23)	1			
BMI	.07 (<.001)	.04 (.006)	.07 (<.001)	1		
Resting heart rate	.03 (.05)	−.19 (<.001)	.23 (.001)	.01 (.39)	1	
Hypertension	.03 (.12)	.01 (.56)	.02 (.27)	.09 (<.001)	−.01 (.36)	1

Abbreviation: TAG-IT-A, Tool for Assessing Glucose ImpairmenT among Adolescents.

^a^ Hypertension was defined as ≥95th percentile adjusted for age, sex, and height or physician diagnosed hypertension.

The variables related to impaired fasting blood glucose included being aged 12 to 14, male, obese, and having a resting heart rate of 70 beats per minutes or higher (Table 3). The odds of having an impaired fasting blood glucose level for each of these variables were at least 1.66 times as great as those of the reference group.

**Table 3:** Odds Ratios and Scores Associated With the Variables Selected for Predicting Elevated Blood Glucose Levels^a^ for TAG-IT-A

Characteristic	OR (95% CI)	TAG-IT-A Score
**Age, y**		
12-14	1.37 (1.07-1.75)	1
15-18	1 [Reference]	0
**Sex**		
Male	2.87 (2.30-3.58)	3
Female	1 [Reference]	0
**BMI, percentile for sex and age^b^**		
≤84th (normal)	1 [Reference]	0
85th-94th (overweight)	1.07 (0.69-1.65)	0
≥95th (obese)	1.66 (1.08-2.55)	2
**Resting heart rate, beats per minute**		
<60	1 [Reference]	0
60-69	1.75 (0.97-3.32)	2
70-79	2.56 (1.44-4.56)	3
80-89	2.83 (1.62-4.95)	3
≥90	3.04 (1.63-5.66)	3

Abbreviations: TAG-IT-A, Tool for Assessing Glucose ImpairmenT among Adolescents; OR, odds ratio; CI, confidence interval; BMI, body mass index.

^a^ Elevated glucose level was defined as ≥100 mg/dL.

^b^ Based on Centers for Disease Control and Prevention growth charts (9).

Twenty-nine percent of the adolescents in NHANES III had a TAG-IT-A score of 3 or higher, and 14% had a TAG-IT-A score of 7 or higher (Table 4). As scores increased from 3 or higher, the sensitivity improved; a TAG-IT-A score of 5 correctly identified 76% of participants with impaired fasting blood glucose .

**Table 4:** Percentage, Sensitivity, and Specificity for Selected Scores of TAG-IT-A^a^

Score (%)	Sensitivity, %	Specificity, %
≥3 (29)	50.0	65.0
≥4 (12)	60.4	49.5
≥5 (13)	76.2	31.5
≥6 (16)	100	0
≥7 (14)	100	0

Abbreviation: TAG-IT-A, Tool for Assessing Glucose ImpairmenT among Adolescents.

^a^ Percentages do not total 100% because all possible scores are not listed.

 The area under the curve was 0.61 for the TAG-IT-A tool compared with 0.55 for BMI alone, which suggests that the TAG-IT-A tool is modestly more useful than BMI alone in identifying adolescents who may need additional evaluation for impaired fasting blood glucose (Figure).

**Figure:**
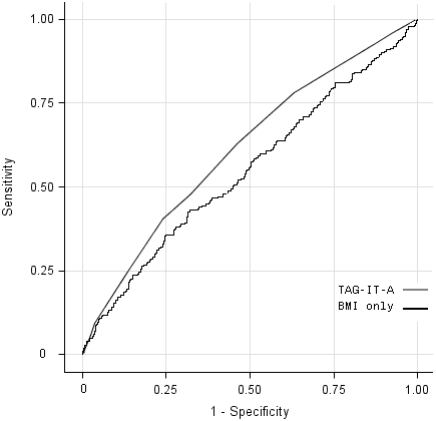
Receiver operating characteristic (ROC) curves using Tool for Assessing Glucose ImpairmenT for Adolescents (TAG-IT-A) and body mass index (BMI) alone with NHANES III data to predict glucose levels ≥100 mg/dL among adolescents. The TAG-IT-A model is a tool developed for use in the community to assess risk of glucose impairment in adolescents. The area under the curve for TAG-IT-A is 0.61 (*P* < .001) and for BMI only is 0.55 (*P* = .10). Abbreviations: ROC, receiver operating characteristic; TAG-IT-A, Tool for Assessing Glucose ImpairmenT among Adolescents; BMI, body mass index; NHANES III, National Health and Nutrition Examination Survey, 1999-1994.

**Table:** 

TAG-IT-A		BMI	
**Sensitivity**	**1-Specificity**	**Sensitivity**	**1-Specificity**
**100.0**	**0**	**100.0**	**0**
**100.0**	**0.5**	**100.0**	**0**
**99.5**	**0.9**	**99.5**	**0.9**
**95.7**	**7.9**	**95.7**	**7.8**
**79.2**	**36.2**	**79.2**	**36.2**
**62.8**	**52.9**	**62.8**	**52.9**
**62.8**	**52.9**	**62.8**	**52.9**
**59.4**	**57.0**	**59.4**	**57.0**
**44.4**	**70.5**	**44.4**	**70.5**
**44.4**	**70.5**	**44.4**	**70.5**
**26.6**	**95.9**	**26.6**	**95.9**
**9.7**	**95.9**	**9.7**	**95.9**
**9.7**	**95.9**	**9.7**	**95.9**
**5.8**	**97.6**	**5.8**	**97.6**

## Discussion

We found that fasting blood glucose levels were positively related to BMI. Eisenmann et al (13) reported that various markers for insulin sensitivity, including fasting blood glucose levels, were higher among obese children than among normal-weight children. We have reported similar findings in our patient population (14). In addition, we found that BMI was positively correlated with hypertension status. Obese girls were 6 times more likely to have hypertension than normal-weight girls (15). Falaschetti et al (16) reported that a 1 kg/m^2^ increase in BMI in children was associated with a 1.4 mm Hg higher systolic blood pressure. To our knowledge, the positive relationship between fasting blood glucose and resting heart rate has not been examined in the adolescent population. Among adults, elevated resting heart rates are linked to higher all-cause and cardiovascular disease mortality (17) and higher odds for elevated blood pressure (systolic and diastolic), abnormal fasting blood glucose, hypertriglyceridemia, and obesity (18). Although only 4% of adolescents in our sample had a heart rate higher than 100 beats per minute, measuring and tracking resting heart rate may be important because those with a resting heart rate above 70 beats per minute were twice as likely to have impaired fasting blood glucose.

Age, sex, BMI, and resting heart rate were the only predictors included in the final model of the TAG-IT-A score. These variables are different from what was included in the adult TAG-IT score. Koopman et al (5) found that, in addition to these 4 predictors, it was necessary to include family history of diabetes and history of hypertension in the model. Our analysis was limited by the lack of available data on family history of diabetes in the NHANES data sets. Including family history of diabetes in the model may have improved its predictive value as measured by ROC analysis (19). Others have developed screening tools for assessing abnormal blood glucose values in adults and reported that in addition to age, BMI, and race, hypertension medication use was also an important predictor (6). A group from Denmark reported that identification of adolescents at risk for developing type 2 diabetes was improved when blood glucose values and triglyceride concentrations were added to systolic blood pressure and parental diabetes (20).

We hypothesized that the TAG-IT-A score would be an effective predictor of impaired fasting blood glucose among adolescents; however, the TAG-IT-A tool was only modestly better than BMI alone (0.61 vs 0.55, respectively). The area under the curve for the impaired glucose tool in adolescents is less than what has been found in adults (0.61 vs 0.74-0.80). One reason this tool may not be as predictive in adolescents is that other variables, such as lipid aerobic fitness, physical activity levels, or puberty and sexual development, may also be important (19-23). Unfortunately, many of these variables were not consistently available in the NHANES data sets for significance testing and inclusion in the model. Also, the goal of this study was to develop a tool for assessing risk of impaired fasting blood glucose that uses noninvasive variables easily measured in nonmedical settings. Although some lipid measures are available in NHANES, they were excluded from inclusion in the model in order to investigate its applicability for community use. Beyond community use, the parameters necessary for calculating the TAG-IT-A score may also be routinely available in electronic medical records that could be programmed to automatically calculate this score for appropriate patients.

A logical question is what TAG-IT-A score public health or medical practitioners should use to determine if an adolescent needs further screening. If a score of 3 is used, then sensitivity is 50%, but sensitivity improves with higher criteria. The economic costs of additional tests need to be considered. Furthermore, health care practitioners need to decide if they want to maximize the number of cases identified correctly (sensitivity); if so, a value of 5 or higher might be used. In contrast, if health care practitioners are more concerned about minimizing the number of false positives, then a lower score with a higher specificity could be used (eg, 3 or higher, 65% specificity).

Few children and adolescents have fasting blood glucose values above 100 mg/dL (13,23,24). For this reason, using fasting blood glucose measurements to screen the entire population of adolescents is not recommended. New tools are needed to help identify at-risk adolescents who may benefit from additional testing to detect abnormal fasting blood glucose concentrations. Community-based screening of adolescents using the TAG-IT-A tool instead of BMI alone may provide a feasible and somewhat more sensitive way of identifying adolescents at increased risk of glucose impairment and in need of further evaluation or interventions. Having easily assessed measures (eg, heart rate and blood pressure) that are related to illness and death in adults (17) suggests that similar measures may indicate health concerns in adolescents as well.

The limitations of this study include the cross-sectional design of NHANES; adolescents who have fasting blood glucose values of 100 mg/dL or higher may not go on to develop type 2 diabetes. Nguyen et al (19), however, reported that even children who had normal values but were at the higher end of the normal range had an increased risk for developing type 2 diabetes as adults. Based on our findings and those of Nguyen et al (19), it appears that a longitudinal study is warranted to evaluate the effectiveness of the TAG-IT-A tool. Finally, we did not perform subgroup analysis by race because of the small sample size of those with impaired fasting blood glucose (only 11% of the total NHANES III adolescent sample). Therefore, it is unknown whether the tool performs similarly in different races. Similar sample-size issues were observed when sex was explored separately.

The TAG-IT-A tool is a simple measure that uses variables that can be obtained in community settings, and it is modestly better than BMI alone in predicting risk for impaired fasting blood glucose. The usefulness of this screening tool for identifying adolescents who have impaired fasting blood glucose should be further evaluated in community-based settings. Longitudinal studies are also needed to determine if adolescents with a high TAG-IT-A score subsequently develop type 2 diabetes in adulthood.
